# Paracetamol modulates biofilm formation in *Staphylococcus aureus* clonal complex 8 strains

**DOI:** 10.1038/s41598-021-84505-1

**Published:** 2021-03-04

**Authors:** Andi R. Sultan, Kirby R. Lattwein, Nicole A. Lemmens-den Toom, Susan V. Snijders, Klazina Kooiman, Annelies Verbon, Willem J. B. van Wamel

**Affiliations:** 1 Department of Medical Microbiology and Infectious Diseases, Erasmus University Medical Center Rotterdam, Rotterdam, The Netherlands; 2 Department of Biomedical Engineering, Thoraxcenter, Erasmus University Medical Center Rotterdam, Rotterdam, The Netherlands; 3 Department of Microbiology, Faculty of Medicine, Hasanuddin University, Makassar, Indonesia

**Keywords:** Microbiology, Biofilms, Bacterial infection, Preclinical research

## Abstract

*Staphylococcus aureus* biofilms are a major problem in modern healthcare due to their resistance to immune system defenses and antibiotic treatments. Certain analgesic agents are able to modulate *S. aureus* biofilm formation, but currently no evidence exists if paracetamol, often combined with antibiotic treatment, also has this effect. Therefore, we aimed to investigate if paracetamol can modulate *S. aureus* biofilm formation. Considering that certain regulatory pathways for biofilm formation and virulence factor production by *S. aureus* are linked, we further investigated the effect of paracetamol on immune modulator production. The in vitro biofilm mass of 21 *S. aureus* strains from 9 genetic backgrounds was measured in the presence of paracetamol. Based on biofilm mass quantity, we further investigated paracetamol-induced biofilm alterations using a bacterial viability assay combined with N-Acetylglucosamine staining. Isothermal microcalorimetry was used to monitor the effect of paracetamol on bacterial metabolism within biofilms and green fluorescent protein (GFP) promoter fusion technology for transcription of staphylococcal complement inhibitor (SCIN). Clinically relevant concentrations of paracetamol enhanced biofilm formation particularly among strains belonging to clonal complex 8 (CC8), but had minimal effect on *S. aureus* planktonic growth. The increase of biofilm mass can be attributed to the marked increase of N-Acetylglucosamine containing components of the extracellular matrix, presumably polysaccharide intercellular adhesion. Biofilms of RN6390A (CC8) showed a significant increase in the immune modulator SCIN transcription during co-incubation with low concentrations of paracetamol. Our data indicate that paracetamol can enhance biofilm formation. The clinical relevance needs to be further investigated.

## Introduction

Healthcare-related infections caused by biofilms formed by *Staphylococcus aureus* have a high mortality (up to 66%) and includes severe chronic infections, such as osteomyelitis and infective endocarditis, and those related to in-dwelling medical devices^1–5^. The unfavorable outcome of biofilm-associated infections has been attributed to the decreased susceptibility of *S. aureus* to antibiotics and host defenses^1,6–10^. Furthermore, it is known that mature *S. aureus* biofilms produce numerous virulence factors that enhance pathogenicity^1,2^, such as staphylococcal complement inhibitor **(**SCIN), Protein A, and thermonuclease, already during the early stages of biofilm formation^11,12^. Biofilm development of *S. aureus* depends on its genetic background^13^ and is highly affected by environmental conditions, including the substrate to which the biofilm is attached to^1,2,13–15^ and growth media composition^12,15,16^.

It has been shown that some compounds and drugs that are often paired with antibiotics can modulate *S. aureus* responses that further reduces antibiotic susceptibility^17,18^. Nonsteroidal anti-inflammatory drugs, like acetylsalicylic acid (aspirin) and ibuprofen, can increase the inhibitory concentration of fusidic acid, an anti-staphylococcal drug^17^. Many other antibiotics used to treat *S. aureus* infections, such as beta-lactams and vancomycin, may actually promote biofilm formation^6–9^. Furthermore, non-antibiotic drugs like acetylsalicylic acid can even modulate biofilm generation^18,19^. Paracetamol, another antipyretic drug, is frequently used before and during early infection symptoms and is often given concomitantly with antibiotic treatment once infection has been confirmed. The mechanism of paracetamol remains uncertain on the molecular level and is different from acetylsalicylic acid in that it does not induce anti-inflammatory effects^20,21^. Until now, it has not been investigated if paracetamol has an influence on biofilm formation and development.

Clinical isolates of *S. aureus* consist of various genetic backgrounds with an unequal worldwide distribution^22^. One of the prominent clonal clusters, clonal complex 8 (CC8), causes a significant proportion of *S. aureus* infections in certain regions^22^. For example, methicillin-resistant USA300 is the most prevalent strain in the United states, while ST-239 is mainly found in Asia, Australia, Eastern Europe, and South America^22^. Considering that the *S. aureus* regulator pathway for biofilm formation and virulence factor production are correlated^23,24^, we also studied the impact of paracetamol on the transcription of staphylococcal complement inhibitor (SCIN) protein. SCIN is a potent immune modulator which is able to inhibit host complement activation pathways during the early stages of biofilm formation^12^.

Since paracetamol is frequently being used in the early stages of infection when biofilms are formed^12^, we studied the effect of paracetamol exposure on *S. aureus* biofilm formation using 21 strains from 9 genetic backgrounds, which includes the clinically relevant CC8 strains ST239 and USA300. In addition, the impact of paracetamol on the transcription of SCIN protein was determined.

## Results

### Clinically relevant doses of paracetamol have limited effect on *S. aureus* planktonic growth

**Figure 1:**
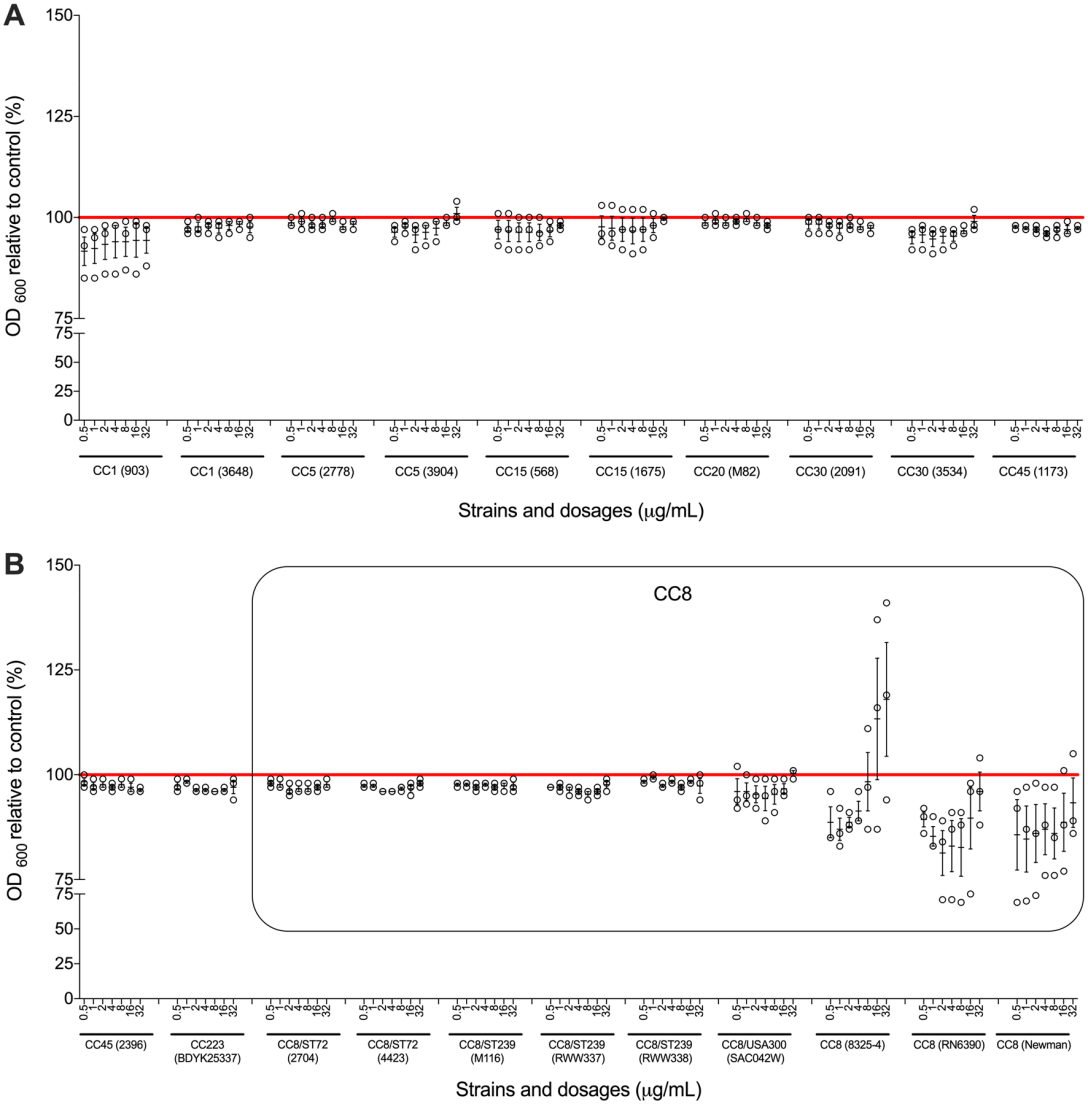
Broth microdilution assays with paracetamol. Broth microdilution susceptibility tests were performed on all study strains. (**A**) Strains with genetic backgrounds of CC1, CC5, CC15, CC20, CC30 and CC45. (**B**) Strains with genetic backgrounds of CC45, CC72, CC223 and CC8. The results are depicted as the percentage of paracetamol treated *S. aureus* MICs relative to its control (untreated). The red horizontal line represents the control. The black box outlines CC8 strains. Each black circle represents one experiment. Data points are presented with SEM of all three separate experiments performed for each dilution.

Since the concentration of paracetamol in human serum is normally below 30 µg/mL^25,26^, we overnight co-incubated *S. aureus* with 0.5, 1, 2, 4, 8, 16, and 32 µg/mL of paracetamol*.* We found that these clinically relevant concentrations of paracetamol had no significant effect on planktonic growth of *S. aureus* strains, except for some of the strains having a CC8 genetic background (Fig. 1). Results in these CC8 strains were variable: the growth rate of strain 8325-4 was higher in the presence of higher doses of paracetamol, while RN6390 and Newman had lower growth rates at higher concentrations.

### Low doses of paracetamol increase biofilm formation of CC8 genetic cluster strains

**Figure 2:**
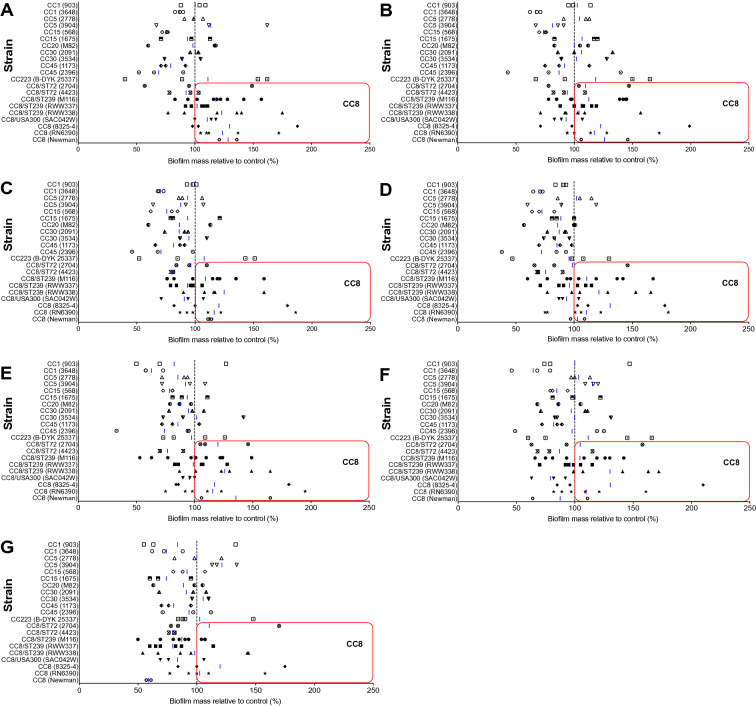
*Staphylococcus aureus* biofilm mass changes in response to various concentrations of paracetamol. Paracetamol stimulates and reduces biofilm formation at clinical doses in strains with different genetic backgrounds, most notably those belonging to CC8 including ST239. Biofilms were grown and co-incubated with 0.5 (**A**), 1 (**B**), 2 (**C**), 4 (**D**), 8 (**E**), 16 (**F**), and 32 (**G**) μg/mL of paracetamol. Red boxes outline the biofilm mass increase of CC8 strains above control mass. Data from at least three separate experiments are presented as aligned dot plots with a blue line indicating the mean value.

To study the effect of paracetamol on biofilm formation, a biofilm quantification assay was performed on all strains following overnight incubation with paracetamol using the same concentrations as used in the planktonic growth experiments. In 15 of the 21 strains, an increase in biofilm mass could be demonstrated (Fig. 2). Of particular note, this phenomenon was primarily observed in *S. aureus* strains belonging to CC8 (2774 (ST72), M116 (ST239), RWW337 (ST239), RWW338 (ST239), SAC042W (USA300), 8325-4, RN6390, and Newman), and less visible in non-CC8 strains (CC1 (903), CC5 (2778, 3904), CC15 (1675), CC30 (2091, 3534), and CC223) when exposed to doses of paracetamol less than 32 µg/mL. Not every paracetamol concentration below 32 µg/mL always led to a clear increase in biofilm mass. For example, the mean value of strain M116 (ST239) indicates biofilm mass increased at 0.5–4 μg/mL and 16 μg/mL, whereas at 8 μg/mL biofilm mass was unaffected. Conversely at these doses, other strains of various backgrounds were observed with mainly reductions in biofilm and as low as obtaining only 63% (mean) of the control biofilm mass for 8 µg/mL paracetamol (strain 3648 (CC1); Fig. 2E).

### Paracetamol did not significantly alter the metabolism rate of biofilm-associated *S. aureus*

**Figure 3:**
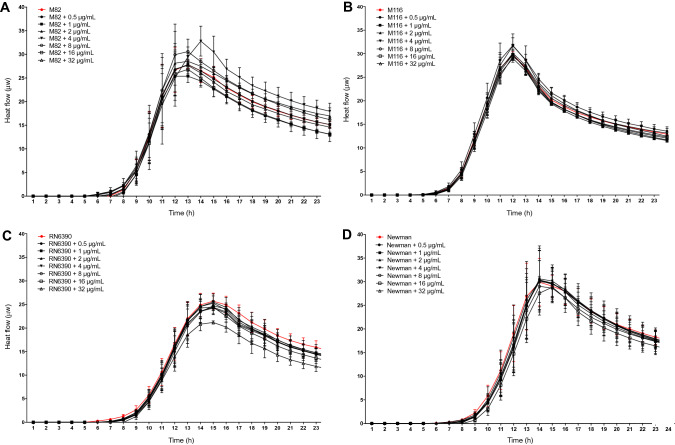
The metabolic rate of biofilms in the presence of different dosages of paracetamol. Metabolic heat flow generated during overnight biofilm formation at 37 °C and co-incubation without (red line) or with 0.5–32 µg/mL paracetamol of *S. aureus* CC20 strain M82 (**A**) and CC8 strains; M116 (**B**), RN6390 (**C**), and Newman (**D**) were monitored with isothermal microcalorimetry in real-time. Data from three separate experiments are presented as mean value and SEM.

To determine if the paracetamol-associated increase in biofilm formation was only due to an increase in the number of bacterial cells, we studied the metabolic rate of *S. aureus* biofilms (CC20 strain M82 and CC8 strains: M116, RN6390, and Newman) when exposed to different concentrations of paracetamol. The addition of paracetamol did not change the heat flow by *S. aureus* during 24 h biofilm formation for all strains tested (Fig. 3)*.* Some minor, non-significant deviations included a delayed heat curve at 4 µg/mL in strain M82 (CC20) (Fig. 3A) and reduced heat flow at the highest therapeutic concentration (32 µg/mL) in strain RN6390 (CC8) (Fig. 3C). These data indicate that although the biomass of biofilms of CC8 strains generally increased when exposed to paracetamol, an increase of bacterial cells is unlikely to be the cause.

### Paracetamol modulates polysaccharide expression during biofilm formation

**Figure 4:**
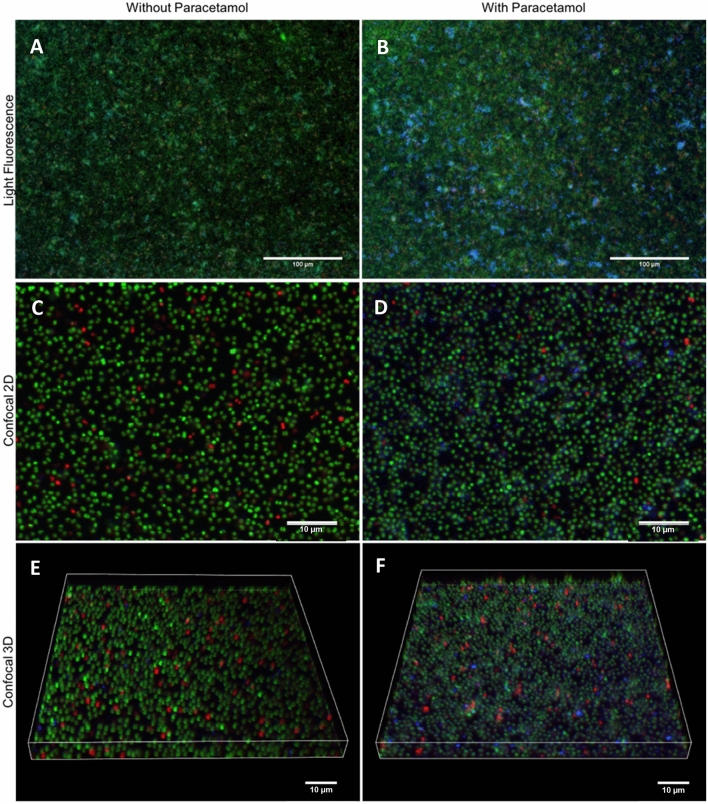
The effect of paracetamol on staphylococcal biofilm matrix composition. Biofilms of *S. aureus* strain M116 (CC8, ST239) were grown at 37 °C without (**A**,**C**,**E**) and with (**B**,**D**,**F**) paracetamol (2 μg/ml). Fluorescent images of stained biofilms were captured using inverted light fluorescence microscopy (**A**,**B**) and confocal laser scanning (**C**–**F**) microscopy with corresponding three-dimensional volume rendering (**E**,**F**) acquired using a Nikon A1R + microscope with NIS-Elements Advanced Research software (Version 4.50; Nikon Instruments). For all images, green fluorescence (SYTO 9 stained) are live bacteria, red fluorescence (PI stained) are dead bacteria and/or eDNA, and blue fluorescence (WGA stained) are N-Acetylglucosamine. All images are representative of three separate experiments.

An explanation for the increase of biofilm mass could be an increase in non-cellular components, such as extracellular DNA (eDNA), proteins and/or polysaccharides^1,27–31^. To investigate this hypothesis, we co-incubated *S. aureus* strain M116 (CC8) biofilms with 2 μg/mL of paracetamol overnight, and studied the biofilms using light fluorescence and confocal microscopy (Fig. 4). Paracetamol-exposed biofilms had an increase of N-Acetyl glucosamine content in their extracellular matrix (Fig. 4).

### Small doses of paracetamol increase immune-modulator transcription

**Figure 5:**
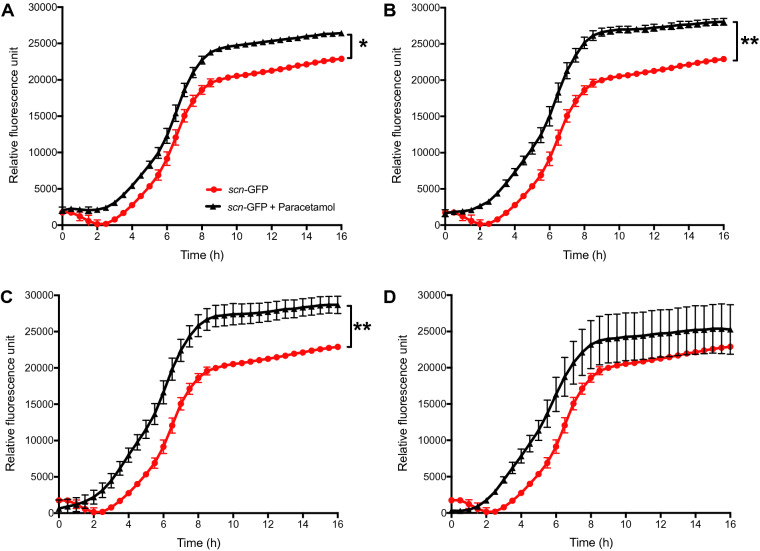
Transcription of the SCIN promoter following paracetamol incubation. Transcription of the SCIN gene promoter—GFP (*scn*-GFP +) in strain RN6390 (CC8) was monitored during overnight incubation at 37 °C without and with 0.5 (**A**), 1 (**B**), 2 (**C**) or 4 (**D**) μg/mL of paracetamol. A single asterisk (p < 0.05) and two asterisks (p < 0.01) represent a statistically significant difference between without and with paracetamol treatment. Data from three separate experiments are presented as mean value and SEM.

RN6390 (CC8) containing a SCIN promoter-green fluorescence protein fusion was used to study the effect of paracetamol exposure on the transcription of the immune modulator SCIN protein. During overnight co-incubation with paracetamol, the biofilms had a significant upregulation of s*cn* promoter transcription (p = 0.009) when lower doses (< 4 µg/mL) of paracetamol were added during biofilm formation (Fig. 5A–C). No difference was observed with a higher dose of 4 µg/mL (Fig. 5D).

## Discussion

In this study, we show that paracetamol exposure can lead to an increase in biofilm mass of *S. aureus* strains, particularly from the CC8 genetic background which includes the highly prevalent ST239 and USA300 strains. Bacterial metabolism during biofilm formation did not significantly increase as a consequence of adding paracetamol. It could be that the total number of biofilm-associated bacteria exposed to paracetamol is less than the untreated bacteria, but those fewer exposed cells are producing more heat due to the increased production of polysaccharides and secreted proteins. However, the heat production due to polysaccharide and secreted protein generation might be negligible in comparison to the heat produced due to cellular growth. Regardless, in both cases there is no increase in the number of biofilm-associated bacterial due to paracetamol treatment and thus, the observed increases of biofilm mass due to paracetamol exposure seems to be best explained by an increase of non-cellular components.

Furthermore, the increase in biofilm mass was associated with an increase in the part of the biofilm rich in N-Acetyl glucosamine, presumably polysaccharide intercellular adhesin (PIA). PIA is a positively charged poly-β(1–6)-*N*-acetylglucosamine (PNAG) and the predominant exopolysaccharide component of the extracellular polymeric matrix of staphylococcal biofilms encapsulating bacteria^30,32^. Additionally, PIA mechanically and chemically prevents the killing of *S. aureus* by phagocytizing leukocytes, antimicrobial peptides, and antibiotics^30,32,33^. No previous data regarding PIA modulation by paracetamol exists to our knowledge. However, a study by Dotto, et al.^18^ showed that acetylsalicylic acid promotes biofilm formation of *S. aureus* in a PIA-dependent manner. These data suggest that use of both paracetamol and acetylsalicylic acid during *S. aureus* infections may be potentially harmful due to increasing biofilm formation.

Bacterial secreted proteins were another consideration in our study. Since the regulator pathway for biofilm formation and toxin production, including immune modulators, are correlated^23,24^, we expected to find that immune modulator production by *S. aureus* could be affected by paracetamol as well. Our data indeed showed that SCIN production can be stimulated significantly by low doses of paracetamol*.* This finding mimics the data from Price et al.^34^, which demonstrated that acetylsalicylic acid could modulate *S. aureus* virulence factor production. SCIN is a potent immune modulator, which is able to inhibit the release of chemoattractant C5a and therefore blocking the host complement activation pathways^35,36^. Previously, we showed that *S. aureus* produced SCIN already during the early stages of biofilm formation^12^. The observed increase in SCIN production, along with PIA and potentially other virulence factors yet to be investigated, in response to paracetamol would aid invading staphylococci to evade the innate immune system and potentiate infection.

Currently, the mechanism of biofilm modulation by paracetamol has not been elucidated, but there are indications that an impaired iron regulation within cells may influence this phenomenon^37,38^, probably via iron chelation by paracetamol^39^. In an iron-restricted condition, biofilm and virulence factor production is increased^18,40^. Paracetamol has been demonstrated in vivo to reduce excess hepatic iron after administration^41^. In addition, a previous study on acetylsalicylic acid and biofilm showed that free Fe^2+^ reduction in culture media by acetylsalicylic acid, via iron chelation, could promote biofilm formation of *S. aureus* CC5 and CC8 strains, including Newman and USA300^18^. This observation suggests that iron-modulation by paracetamol may enhance *S. aureus* biofilm formation.

In summary, this study indicates that current clinical concentrations of an analgesic-antipyretic like paracetamol may have a role in the development and persistence of *S. aureus* biofilm-related infections, especially, but not limited to, strains belonging to CC8. For clinical practice our data suggest that in patients with a suspected *S. aureus* infection, the indication for paracetamol administration should be carefully weighed against the risk of increased biofilm formation. The mechanism of action and the effect on an established, mature biofilm by paracetamol need to be investigated in future studies.

## Materials and methods

### Bacterial strains and growth condition

The *S. aureus* strains used in this study are listed in Table 1. Strains were plated on blood agar (5% sheep blood; BD, Trypticase, Thermo Fisher Scientific, Bleiswijk, the Netherlands) and incubated overnight at 37 °C. The green fluorescent protein (GFP) construct containing strains were plated on tryptic soy agar (TSA) supplemented with 10 µg/ml chloramphenicol (Oxoid Limited, Basingstoke, UK).

**Table 1:** *S. aureus* strains used in this study.

Strain	Genetic background	Description	Source/Ref
Mup903	CC1	Commensal strain	^48^
Mup3648	CC1	Commensal strain	^48^
Mup2778	CC5	Clinical strain	^48^
Mup3904	CC5	Clinical strain	^48^
Mup568	CC15	Clinical strain	^48^
Mup1675	CC15	Clinical strain	^48^
M82	CC20	Clinical strain	^12^
Mup2091	CC30	Clinical strain	^48^
Mup3534	CC30	Clinical strain	^48^
Mup1173	CC45	Clinical strain	^48^
Mup2396	CC45	Clinical strain	^48^
B-DYK 25337	CC223	Clinical strain	Erasmus MC
Mup2704	CC8, ST72	Clinical strain	^48^
Mup4423	CC8, ST72	Commensal strain	^48^
M116	CC8, ST239	Clinical strain	^12^
RWW337	CC8, ST239	Clinical strain	^49^
RWW338	CC8, ST239	Clinical strain	^49^
SAC042W	CC8, USA300	SAC042W, Clinical strain	^50^
8325-4	CC8	Laboratory strain	^51^
RN6390	CC8	Laboratory strain	^52^
Newman	CC8	Laboratory strain	^53^
*scn*-GFP ( +)	CC8	*scn* promoter (pSK236GFP in RN6390)	^54^
PrP-GFP ( +)	CC8	Page repressor promoter (pACL1484 in RN6390)	^47^

### The effect of paracetamol on planktonic bacteria

To determine the effect of paracetamol (Sigma Aldrich, Zwijndrecht, the Netherlands) on bacterial growth, a broth microdilution method was performed for all strains^41,42^. One hundred microliter of a 1:100 dilution (optical density (OD) of 0.50 (± 0.05) at 600 nm in NaCl 0.9%) of each strain in Iscove`s Modified Dulbecco`s Medium (IMDM) (Gibco^®^, Thermo Fisher Scientific) was added to 100 µl of culture media containing paracetamol for final concentrations of 0.5, 1, 2, 4, 8, 16, and 32 µg/mL. After 24 h incubation at 37 °C in sterile round-bottom 96-well polystyrene tissue culture plates (Costar no. 3799; Corning Inc., Corning, NY), the optical densities (OD_600nm_) were read in a microplate reader (Epoch 2 Microplate reader, BioTek Instruments, Inc., Winooski, VT, USA).

### Biofilm formation mass assessment

In vitro biofilms were generated from both wild type and GFP-containing strains (Table 1) by the method described previously^12^. Biofilm formation was assessed using a dynamic, microtiter plate biofilm formation assay as previously described by Christensen et al.^2,12,43,44^, with slight modifications. Briefly, for each strain or isolate, 5 mL of NaCl 0.9% was inoculated with overnight grown *S. aureus* (at 37 °C on blood agar) until an OD_600nm_ of 0.50 (± 0.05) was reached. Thereafter, 10 µL was dispensed into a sterile flat-bottom 96-well polystyrene tissue culture plates (Costar no. 3596; Corning Inc., Corning, NY) containing a serial dilution (0.5, 1, 2, 4, 8, 16, and 32 μg/mL) of paracetamol in 190 µL of IMDM. The plates were incubated at 37 °C for 24 h with continuous shaking at 150 rpm. Bacterial growth was then measured using a microplate reader (BMG technologies, Ortenberg, Germany) at OD_600nm_. Afterwards, the visible biofilms which had formed in the wells were washed once with 200 µL of sterile phosphate-buffered saline (PBS) containing 1% BSA and 0.05% azide. Biofilms were then air dried and stained with 50 µL of 1% crystal violet in distilled water for 2 min. Excess crystal violet was removed by washing the plates with distilled water for five times. The stained biofilm was then dissolved in 200 µL of extraction solution (50% dH_2_O, 40% EtOH, 10% acetyl acid) and the absorbance of crystal violet measured at OD_490nm_ in the Epoch 2 microplate reader.

### Fluorescence staining of biofilm

For visualization of the cellular and extracellular matrix components, biofilms were stained using a LIVE/DEAD BacLight Bacterial Viability Kit (Thermo Fisher Scientific) and Wheat Germ Agglutinin (WGA)—Alexa Fluor 350 conjugate (Invitrogen BV, Breda, the Netherlands), according to the manufacturer’s protocol with slight modification. Co-incubating biofilms with 2 μg/mL of paracetamol was chosen based on biofilm mass increases observed occurring at 0.5–4 μg/mL and consideration of the in vivo situation, which includes a paracetamol peak serum concentration of 20–30 μg/mL (> 30 is considered toxic), 10–20% bound to red blood cells, plasma half-life of 1.5–2.5 h, and 90–95% of the standard dose is conjugated into inactive metabolites subsequently excreted in the urine^45^. Follow incubation, biofilms were washed once with 200 µL of IMDM. Then 50 µL of IMDM, 50 µL of 15 μM propidium iodide (PI), 50 µL of 2.5 μM SYTO9, and 0.5 µL of 1 mg/mL WGA-Alexa fluor 350 conjugate was added to each well and the plate incubated at 22 °C on an orbital shaker (300 rpm) in the dark for 35 min. The biofilms were then imaged using an Olympus IX51 fluorescence microscope (Olympus Nederland B.V., Zoeterwoude, the Netherlands) with 20- and 40-times magnification. For confocal microscopy, a custom-built Nikon A1R + confocal microscope^46^ (Nikon Instruments Europe, Amsterdam, the Netherlands) was used with a 60-times water immersion lens (CFI NIR Apo 60X W, Nikon Instruments). For imaging, WGA-Alexa fluor 350 conjugate was excited at 405 nm, detected at 450/50 nm (center wavelength/bandwidth), Syto9 was excited at 488 nm, detected at 525/50 nm, and PI was excited at 561 nm, detected at 595/50 nm, and^4^ DiD excited at 640 nm. Biofilms imaged with the confocal microscope were grown in CLINIcell culture chambers (CLINIcell25–50-T, REF 00106, MABIO, Tourcoing, France) in 12 mL using the same biofilm formation and fluorescence staining methods with volumes proportionally adjusted according to manufacturer protocol.

### Bacterial metabolism rate measurement

The effect of paracetamol on bacterial metabolism was monitored with a microcalorimeter according to the previously described protocol^11^. Briefly, overnight cultures of *S. aureus* on blood agar at 37 °C were suspended in 5 mL NaCl 0.9% until OD_600nm_ of 0.50 (± 0.5) was reached. Then 10 µL of this suspension was mixed with 9990 µL IMDM to create a 1:1000 dilution. Ten µL of the diluted suspension was added into sterile plastic “insert” tubes (designed for the microcalorimeter) containing 190 µL of different concentrations (0.5–32 mg/mL) of paracetamol in IMDM. These tubes were then placed into sealed platinum tubes and placed inside a multi-channel microcalorimeter (calScreener SymCel, Sverige AB, Sweden) for 24 h at 37 °C to determine real-time bacterial metabolic activity.

### Measurement of immune modulators SCIN transcription

To study the effect of paracetamol on the transcription of the immune modulator SCIN during biofilm formation, RN6390 carrying a *scn* (SCIN) promotor GFP construct and RN6390 carrying a plasmid that constitutively produces GFP as a positive control (phage repressor promoter-GFP fusion construct) were used as previously described by Rooijakkers et al.^47^ (Table 1). In vitro biofilms were generated from both GFP-containing strains (Table 1) by the method described previously^12^. After an hour of incubation at 37 °C, the growth medium was replaced with 200 µL of fresh IMDM medium containing paracetamol (serial dilution 0.5–32 μg/µL). The biofilms were then incubated in a FLUOstar Optima microplate fluorescence reader (BMG Lab Technologies, Chicago, IL, USA) at 37 °C with 150 rpm periodic rotational shaking. The accumulation of fluorescence, used as a measure for gene transcription, was determined (excitation at 485 nm, emission at 520 nm, and gain setting 1738) automatically every 5 min during the 4th and 5th hours of biofilm formation. Median fluorescence intensities (MFI) of the *scn* promoter-GFP strain co-incubated with paracetamol were compared to the MFI of the strain incubated without paracetamol.

### Data analysis

Confocal image acquisition and analysis were obtained using Nikon Instruments Software (NIS)-Elements Advanced Research (Version 4.50; Nikon Instruments). Statistical analysis and data graphical representation was performed with GraphPad Prism (GraphPad Software Inc., Version 5.01 for Windows, San Diego, CA, USA, www.graphpad.com) and Microsoft Excel (Microsoft Corporation, 2010, https://office.microsoft.com/excel). Data were analyzed with an unpaired t-test or one-way ANOVA; a P-value ≤ 0.05 was considered as statistically significant. All experiments were independently repeated at least three times and data are presented as mean (SEM) or as median with range.

## Data Availability

The data that support the findings of this study are available from the corresponding author upon reasonable request.
